# Introduction to Bionanocomposites

**DOI:** 10.1039/d3na90115g

**Published:** 2024-01-15

**Authors:** Sabu Thomas, Maya Jacob John, Aji P. Mathew

**Affiliations:** a Mahatma Gandhi University Kottayam Kerala India; b Council for Scientific and Industrial Research (CSIR) Pretoria South Africa; c Stockholm University Sweden

## Abstract

Sabu Thomas, Maya John and Aji Mathew introduce the *Nanoscale Advances* themed issue on Bionanocomposites.
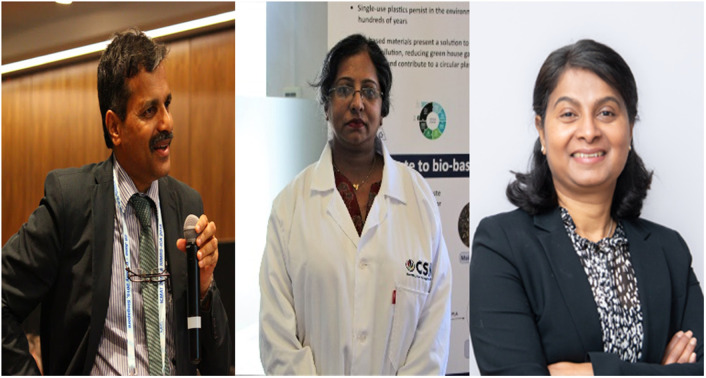

Bionanocomposites comprising biobased polymers and nanosized bio-based fillers are novel materials with tunable properties and have diverse applications in packaging, environmental remediation and the biomedical sector. Bionanocomposite materials also have a significant role to play in the implementation of a functional circular economy.

In this themed issue, leading researchers from academia and industry were invited to submit reviews or their latest research on topics aligned to the development of bionanocomposites from renewable resources. Studies dealing with waste conversion to bio-based products and the development of bionanocomposites have been included in this issue. This issue consists of 7 research articles.

As guest editors of this themed issue, we acknowledge all the authors and reviewers who have contributed to its publication. We would also like to thank the technical support team at the Royal Society of Chemistry for their assistance in preparing this themed issue.

## Supplementary Material

